# Necrosulfonamide exerts neuroprotective effect by inhibiting necroptosis, neuroinflammation, and α-synuclein oligomerization in a subacute MPTP mouse model of Parkinson’s disease

**DOI:** 10.1038/s41598-023-35975-y

**Published:** 2023-05-31

**Authors:** Yea-Hyun Leem, Do-Yeon Kim, Jung-Eun Park, Hee-Sun Kim

**Affiliations:** 1 Department of Molecular Medicine and Inflammation-Cancer Microenvironment Research Center, School of Medicine, Ewha Womans University, 808-1 Magok-Dong, Gangseo-gu, Seoul, 07804 South Korea; 2 Department of Brain and Cognitive Sciences, Ewha Womans University, Seoul, South Korea

**Keywords:** Neuroscience, Diseases

## Abstract

Parkinson’s disease (PD) is an incurable movement disorder characterized by dopaminergic cell loss, neuroinflammation, and α-synuclein pathology. Herein, we investigated the therapeutic effects of necrosulfonamide (NSA), a specific inhibitor of mixed lineage kinase domain-like protein (MLKL), in a subacute 1-methyl-4-phenyl-1,2,3,6-tetrahydropyridine (MPTP) mouse model of PD. MLKL is an executor of necroptosis, a programmed cell death pathway that causes inflammation. Repeated administration of NSA resulted in the recovery of impaired motor performance and dopaminergic degeneration. Furthermore, NSA inhibited the phosphorylation, ubiquitylation, and oligomerization of MLKL, all of which are associated with MLKL cell death-inducing activity in dopaminergic cells in the substantia nigra (SN). NSA also inhibited microglial activation and reactive astrogliosis as well as the MPTP-induced expression of proinflammatory molecules such as tumor necrosis factor-α, interleukin-1β, inducible nitric oxide synthase, and cystatin F. Furthermore, NSA inhibited α-synuclein oligomerization and phosphorylation in the SN of MPTP-treated mice by inhibiting the activity of glycogen synthase kinase 3β and matrix metalloproteinase-3. In conclusion, NSA has anti-necroptotic, anti-inflammatory, and anti-synucleinopathic effects on PD pathology. Therefore, NSA is a potential therapeutic candidate for PD.

## Introduction

Necroptosis is a type of programmed cell death implicated in various pathological conditions, including infection, inflammation, ischemic injury, spinal cord injury, and neurodegeneration^1–4^. Necroptosis is indicated by plasma membrane disruption and leakage of damage-associated molecular patterns (DAMPs), such as the high mobility group box 1 protein and mitochondrial DNA, which can lead to a robust immune response and inflammation^5–7^. Receptor-interacting protein kinase (RIPK)1 and RIPK3 and mixed lineage kinase domain-like protein (MLKL) are core mediators in necroptosis, in which the final executor MLKL activated by RIPK3 undergoes oligomerization and membrane targeting in necroptotic signaling^8–10^. MLKL activity is modulated by post-translational modifications (PTMs) such as phosphorylation and ubiquitylation, which are strictly regulated to avoid unnecessary or unintended tissue damage and pathology^11–13^.

Recent studies have demonstrated that necroptosis participates in the pathogenesis of several neurological disorders, including Alzheimer’s disease^14–16^, stroke^17–19^, spinal cord injury^20,21^, and Parkinson’s disease (PD)^22,23^. PD is a movement disorder characterized by the progressive loss of dopaminergic neurons, the accumulation of α-synuclein-insoluble inclusions, and persistent neuroinflammation in the nigrostriatal dopaminergic circuit^24–26^. While its prevalence is increasing worldwide^27^, PD is incurable owing to the lack of efficient and secure therapies. Previous studies have reported that pharmacological inhibition of RIPK1 by necrostatin-1 (Nec-1) or Nec-1 stable (Nec-1s) showed neuroprotective effects in 6-hydroxydopamine (OHDA)-treated PC12 cells, neural cells derived from induced pluripotent stem cells of PD patients with OPA1 gene mutations, and in 1-methyl-4-phenyl-1,2,3,6-tetrahydropyridine (MPTP)-induced PD mice^22,28^. More recently, genetic ablation of *ripk3* or *mlkl* reduced dopaminergic degeneration in OHDA- or MPTP-treated mice^23,29^. These findings strongly indicate the involvement of necroptosis in PD pathogenesis. Thus, controlling necroptotic cell death is considered an appealing strategy for preventing dopaminergic degeneration during PD progression.

Necrosulfonamide (NSA) is a specific MLKL inhibitor that suppresses necroptosis^9^. Recent studies have shown that NSA has a therapeutic window in rodent models of neurological disorders. Besides suppressing MLKL activity, NSA ameliorated neurological impairment by improving antioxidant capacity in a mouse model of spinal cord injury^30^. NSA also alleviated amyloidopathy and tauopathy in a rat model of Alzheimer’s disease^31^. Furthermore, NSA exhibited neuroprotective effects after ischemic brain injury in mice by inducing MLKL ubiquitination and degradation^32^. Moreover, NSA alleviated acute brain injury in a mouse intracerebral hemorrhage model by inhibiting inflammation and necroptosis^4^. However, the potential effect of NSA on the pathophysiology of PD has not yet been reported.

To test an available option for the amending role of NSA in PD pathology, we empirically investigated the effects of NSA on neuroinflammation, α-synuclein pathology, dopaminergic degeneration, and neurobehavioral outcomes in a PD mouse model.

## Materials and methods

### Reagents and antibodies

Necrosulfonamide (NSA) was obtained from Merck Millipore (Billerica, MA). MPTP was purchased from Tokyo Chemical Industry Co. Ltd. (Tokyo, Japan). Avidin–biotin horseradish peroxidase (HRP) complex reagent, biotinylated secondary antibodies, diaminobenzidine tetrahydrochloride, and antifade reagent were obtained from Vector Laboratories (Burlingame, CA, USA). Thioflavin S was obtained from Sigma-Aldrich (St. Louis, MO, USA). The primary antibodies used were as follows: anti-TH (Cat# 58844), anti-TNF-α (Cat# 11948), anti-p-α-synuclein (Ser129, Cat# 23706), anti-α-synuclein (Cat# 2642), ant-MLKL (Cat# 37705), anti-pMLKL (Cat# 37333), anti-RIPK1 (Cat# 3493), anti-RIPK3 (Cat# 15828), anti-ubiquitin (Cat# 3936), and anti-GSK3β (Ser9, Cat# 9336) antibodies from Cell Signaling Technology, Inc. (Danvers, MA, USA); anti-CST7 antibody (Cat# MBS9609742) from Mybiosource Inc. (San Diego, CA, USA); anti-p-GSK3β (Y216, Cat# 612312) and anti-iNOS (Cat# 610432) from BD Bioscience (San Jose, CA, USA); anti-IL-1β (Cat# AF-401-NA) from R&D systems, Inc. (Minneapolis, MN, USA); anti-β-actin (Cat# A1978) from Sigma-Aldrich (St. Louis, MO, USA); Iba-1 from Wako (Osaka, Japan); anti-A11 from Invitrogen (Waltham, MA, USA); anti-GFAP (Cat# 300-141) from Novus Biologicals (Centennial, CO 80112, USA); and anti-S100β from Santa Cruz Biotechnology (Cat# SC-393919; Dallas, TX, USA). HRP-conjugated secondary antibodies were purchased from Bio-Rad (Hercules, CA, USA).

### Animals

Adult male C57BL/6 mice (7 weeks old) were purchased from Orient Bio, Inc. (Seongnam, South Korea), a branch of the Charles River Laboratories. The mice were maintained at 21 °C under a 12-h light: dark cycle and had ad libitum access to water and rodent chow. Every effort was made to minimize animal suffering. All experiments were performed in accordance with the National Institutes of Health and Ewha Womans University guidelines for laboratory animal care and use, and the study was approved by the Institutional Animal Care and Use Committee of the Medical School of Ewha Womans University (#EUM 20-022). The study was carried out in compliance with the ARRIVE guidelines.

### MPTP administration and experimental procedure

**Figure 1:**
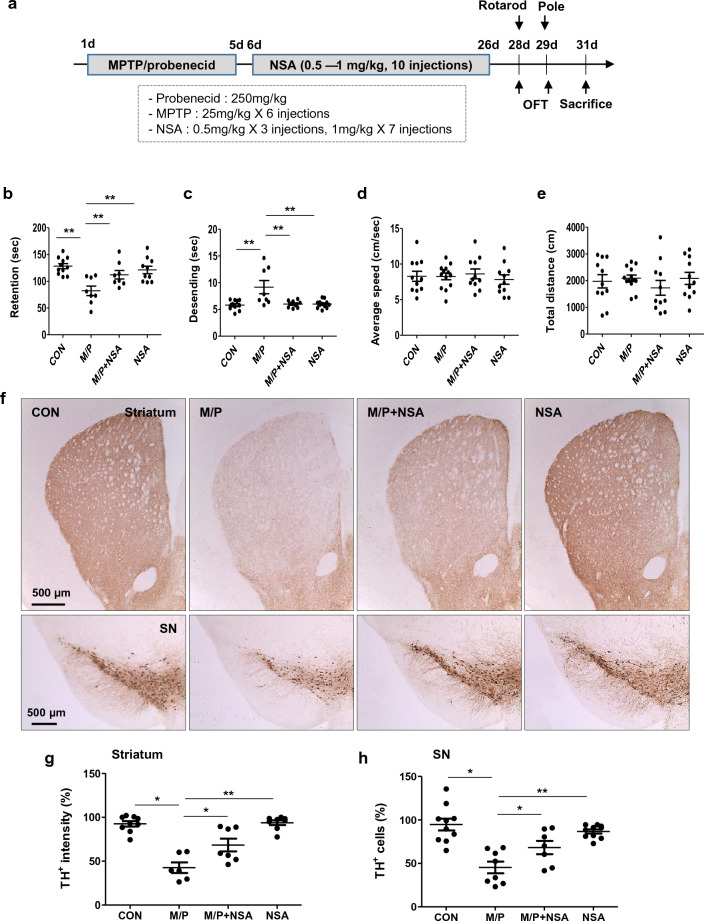
NSA recovered the impaired motor performance and dopaminergic degeneration in MPTP-treated mice. (**a**) Scheme of the experimental procedure. Mice were intoxicated with MPTP for five days and then treated with NSA (0.5 mg/kg/day for three days and 1 mg/kg/day for seven days, every second day of i.p. injection), followed by behavioral tests and sacrifice. (**b**–**e**) NSA produced the delayed retention time in the rotarod test (**b**) and shortening of the descending time in the pole test (**c**) (CON N = 11; M/P N = 8; M/P + NSA N = 8; NSA N = 10). (**d**,**e**) Locomotor activity was not affected by MPTP and/or NSA (CON N = 11; M/P N = 13; M/P + NSA N = 11; NSA N = 11). (**f**–**h**) NSA restored the TH^+^ fibers in the striatum and the TH^+^ cells in the SN in MPTP-treated mice (**f**). The diagram shows the quantification for TH^+^ intensity and cells (**g**,**h**; CON N = 9; M/P N = 6; M/P + NSA N = 7; NSA N = 8). Data are expressed as mean ± SEM. *p < 0.05, **p < 0.01.

The mice were randomly divided into four groups: CON, control; M/P, MPTP + probenecid; M/P + NSA; and NSA (CON, N = 49; M/P, N = 50; M/P + NSA, N = 50; NSA, N = 49). MPTP and probenecid were administered intraperitoneally for five consecutive days. In brief, mice were administered MPTP (25 mg/kg i.p.) twice a day with a 6-h interval on day 1, and once a day on days 2–5. The mice were treated with probenecid (250 mg/kg/day, s.c.) 30 min before MPTP injection to promote the prolonged neuronal retention of MPTP. Thereafter, NSA was intraperitoneally injected for three weeks (0.5 mg/kg/day for three days and 1 mg/kg/day for seven days every two days), and the animals were subjected to behavioral tests two and three days after the last NSA treatment. The mice were decapitated two days after the completion of the behavioral tests (Fig. 1a).

### Behavioral tests

An accelerated rotarod test was performed to assess motor coordination in the mice. Five days before drug treatment, all mice were trained on the rotarod (4–13 rpm) until they remained on the apparatus for 300 s without falling. The rotarod was accelerated from 4 to 40 rpm over a 300-s period. The mice were subjected to three trials at 15-min intervals. The retention times on the rods in each trial were recorded. To evaluate akinesia, a pole test was implemented^33^. The time taken for each mouse to descend the pole was recorded. Each mouse was subjected to three trials, and the average was recorded. For the open field test (OFT), mice were placed in the center of a clear Plexiglas box made of white nonporous plastic (50 cm × 50 cm × 38 cm). The total distance traveled and velocity within 5 min were measured using the EthoVision 17 program (Noldus, Wageningen, Netherlands).

### Histological analysis

Brain sections were obtained according to a previously described method (striatum: every six sections/brain; substantia nigra [SN]: every three sections/brain)^33^. The sections were subjected to endogenous peroxidation inactivation with 3% hydrogen peroxide, and non-specific binding was blocked with 4% bovine serum albumin. The sections were incubated overnight with primary antibodies and then with biotinylated secondary antibodies for 1 h at 25 °C on the following day. The sections were subsequently incubated with an avidin–biotin-HRP complex reagent solution for 1.5 h, and a peroxidase reaction was performed using diaminobenzidine tetrahydrochloride. For double immunofluorescence, non-specific binding was blocked, and the sections were incubated with primary antibodies, followed by fluorochrome-conjugated secondary antibodies. For thioflavin-S (Thio-S) staining, sections were incubated with 0.005% Thio-S for 8 min, washed twice with 50% ethanol for 5 min, and washed with phosphate-buffered saline. The tissue was then mounted using an antifade reagent (Vector Laboratories). Digital images of immunohistochemical and immunofluorescence staining were captured using a Leica DM750 microscope (Leica Microsystems). Quantification was performed using the ImageJ software (NIH, Bethesda, MD, USA).

### Western blot analysis

Tissues collected from the striatum and SN were homogenized in an ice-cold lysis buffer^34^. Subsequently, the samples were vortexed vigorously at 10-min intervals and then incubated for 30 min at 4 °C. The samples were then centrifuged at 20,000×*g* for 30 min, and the supernatant was collected (S1). The pellet was resuspended in lysis buffer, and the supernatant (S2) was obtained by repeated centrifugation. These protein extracts (S1 and S2) represented the Triton X-100-soluble fraction. Protein samples (50–100 μg) were separated by SDS-PAGE, transferred to a nitrocellulose membrane, and incubated with primary antibodies according to the manufacturer’s instructions. To detect MLKL tetramer, the final pellet (insoluble fraction) obtained after S1 and S2 fractionation was resuspended through sonication in 2% SDS lysis buffer, and the lysate was loaded with non-reducing sample buffer. The membranes were thoroughly washed with TBST and incubated with horseradish peroxidase-conjugated secondary antibodies. Subsequently, the blots were developed using an enhanced chemiluminescence detection kit (Thermo Fisher Scientific, Waltham, MA, USA). Using ImageJ software, the density of specific target bands was normalized against that of β-actin for quantification.

### Co-immunoprecipitation assay

Immunoprecipitation of pMLKL was performed according to the manufacturer’s instructions (Dynabeads Protein G Immunoprecipitation Kit; Cat# 10007D; Thermo Fisher Scientific, Waltham, MA, USA). Four samples were pooled per group (N = 4). The tissue samples were homogenized in RIPA lysis buffer (20 mM Tris (pH 7.5), 150 mM NaCl, 1% Triton X-100, 0.1% SDS, 0.5% sodium deoxycholate, and a proteinase inhibitor cocktail). Anti-pMLKL (3 μg) antibodies were captured through incubation with binding buffer containing the magnetic bead for 6 h at 4 °C. The immune complexes were incubated with cell lysates (1 mg) from the SN overnight at 4 °C. Protein G complexes were precipitated, washed three times in washing buffer, and eluted through boiling in the elution buffer and 5 × reducing SDS sample buffer for 10 min at 95 °C. Finally, the immunoprecipitated samples were immunoblotted using an anti-ubiquitin antibody (1:500) with 10% SDS-PAGE.

### Statistical analysis

Statistical analyses were performed using SPSS for Windows (version 18.0; SPSS Inc., Chicago, IL, USA). Differences among groups were analyzed using one-way analysis of variance. Post hoc comparisons were conducted using Tukey’s test. Pearson’s correlation coefficient was used to analyze the correlations between variables. All values were presented as the means ± standard errors of the mean (SEMs). At *p* < 0.05, differences were considered statistically significant.

## Results

### NSA restored dopaminergic degeneration in nigrostriatal pathway and improved behavioral outcomes

To examine the effects of NSA on dopaminergic degeneration in the striatum and SN and motor performance in a PD mouse model, mice were injected with MPTP for 5 days and then treated with NSA (0.5 mg/kg/day for 3 days and 1 mg/kg/day for 7 days, every second day of i.p. injection), followed by behavioral tests and sacrifice, as shown in Fig. 1a. Motor coordination and akinesia were assessed using accelerating-speed rotarod and pole tests to investigate the motor function-restoring role of NSA in the MPTP-treated mice. In the rotarod test, the retention time on the rod was significantly lengthened by NSA in MPTP-treated mice (Fig. 1b; F_3,34_ = 8.03, *p* < 0.01). In the pole test, NSA shortened the delayed descent time in the MPTP-treated mice (Fig. 1c; F_3,34_ = 7.90, *p* < 0.01). In the open field test (OFT), no significant difference in the total distance traveled (Fig. 1d, F3,42 = 0.58, *p* > 0.05) and average velocity (Fig. 1e, F3,42 = 0.26, *p* > 0.05) between the groups was noted, indicating that locomotor activity was not affected by MPTP or NSA treatment in our experimental paradigm. In accordance with the behavioral results, NSA induced the recovery of nigrostriatal degeneration in response to MPTP toxicity, as indicated by the recovery of TH^+^ fibers in the striatum (Fig. 1f,g; F_3, 28_ = 24.07, *p* < 0.05) and TH^+^ cells in the SN (Fig. 1f,h; F_3, 28_ = 13.65, *p* < 0.05). These results imply that NSA is capable of restoring motor performance defects, striatal TH + fiber deficiency, and TH + cell loss in a mouse model of PD.

### NSA inhibited MLKL expression, oligomerization, and phosphorylation in the SN region of MPTP-treated mice

**Figure 2:**
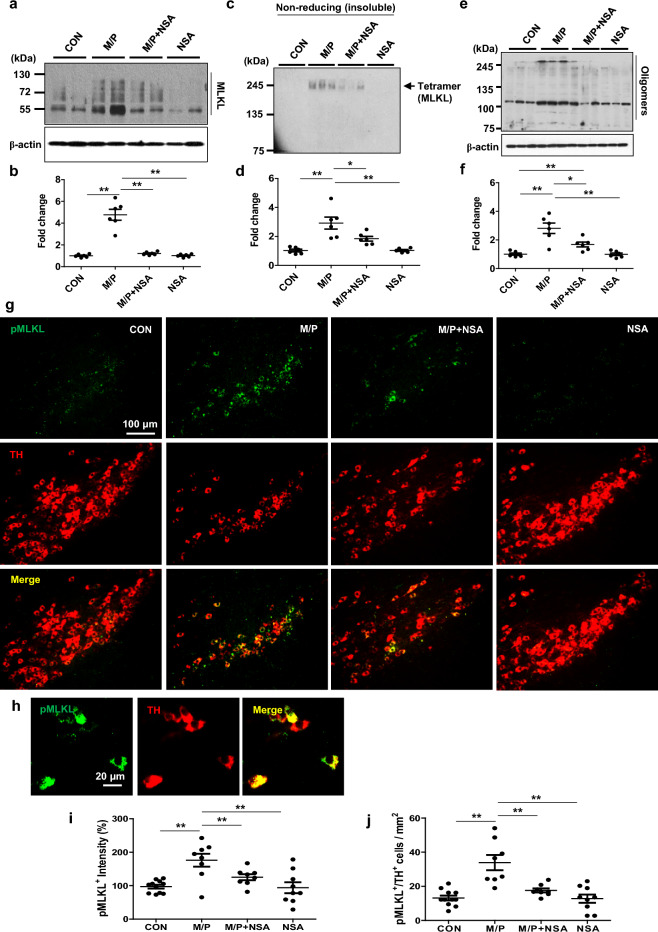
NSA inhibited the expression/oligomerization of MLKL and diminished the pMLKL-expressing dopaminergic neurons in MPTP-treated mice. (**a**) NSA attenuated the MLKL levels in the SN region of MPTP-treated mice. (**b**) The quantification diagram for MLKL levels (CON N = 6; M/P N = 6; M/P + NSA N = 6; NSA N = 6). (**c**) NSA decreased the MLKL tetramer levels in the SN region of MPTP-treated mice. (**d**) The quantification diagram for MLKL tetramer levels (CON N = 6; M/P N = 6; M/P + NSA N = 6; NSA N = 6). (**e**) NSA decreased the MLKL oligomer levels in the SN region of MPTP-treated mice. (**f**) Quantification diagram for MLKL oligomer levels (CON N = 6; M/P N = 6; M/P + NSA N = 6; NSA N = 6). (**g**) NSA reduced pMLKL^+^ intensity and pMLKL^+^/TH^+^ cells in the SN area of MPTP-treated mice. (**h**) A photomicrograph of pMLKL^+^/TH^+^ cells at high magnification. (**i**,**j**) A diagram quantifying the pMLKL^+^ intensity and pMLKL^+^/TH^+^ cells (CON N = 11; M/P N = 8; M/P + NSA N = 8; NSA N = 9). Data are expressed as mean ± SEM. *p < 0.05, **p < 0.01.

Next, we verified the involvement of necroptotic signaling in dopaminergic neuronal cell death, and the potential role of NSA in the SN of a subacute MPTP mouse model of PD. Since MLKL undergoes oligomerization and phosphorylation during necroptotic signaling^9,12^, we examined the effect of NSA on the PTMs of MLKL. MLKL protein expression significantly increased in response to MPTP toxicity, which was mitigated by NSA treatment (Fig. 2a,b; F_3, 20_ = 52.80, *p* < 0.01). The smeared bands appear to be the ubiquitylated form of MLKL. In the insoluble fraction, MLKL tetramer expression was increased by MPTP and inhibited by NSA (Fig. 2c,d; F_3, 20_ = 15.36, *p* < 0.01). Accordingly, western blot analysis using a conformation-sensitive A11 antibody (specifically recognizing oligomeric proteins) revealed that MPTP treatment increased MLKL oligomer formation, which was reduced by NSA (Fig. 2e,f; F_3, 20_ = 16.35, *p* < 0.01). The expression of RIPK1 and RIPK3 proteins was significantly enhanced in the SN region of MPTP mice, but NSA did not reduce these levels (Fig. S1a,b). Moreover, pMLKL^+^ (Ser345-p) intensities and the number of pMLKL^+^/TH^+^ cells in the MPTP group were higher than those in the control group, and NSA treatment significantly reduced the MPTP-induced increase in the pMLKL population (Fig. 2g–j; pMLKL^+^, F_3, 32_ = 8.28, *p* < 0.01; pMLKL^+^/TH^+^, F_3, 32_ = 14.37, *p* < 0.01). These findings imply that dopaminergic cell loss may be partly attributed to necroptosis and that NSA may restrain the ability of MLKL to transduce necroptotic signaling by inhibiting the expression, oligomerization, and phosphorylation of MLKL.

### NSA interrupted the ubiquitylation of MLKL in the SN region of MPTP-treated mice

**Figure 3:**
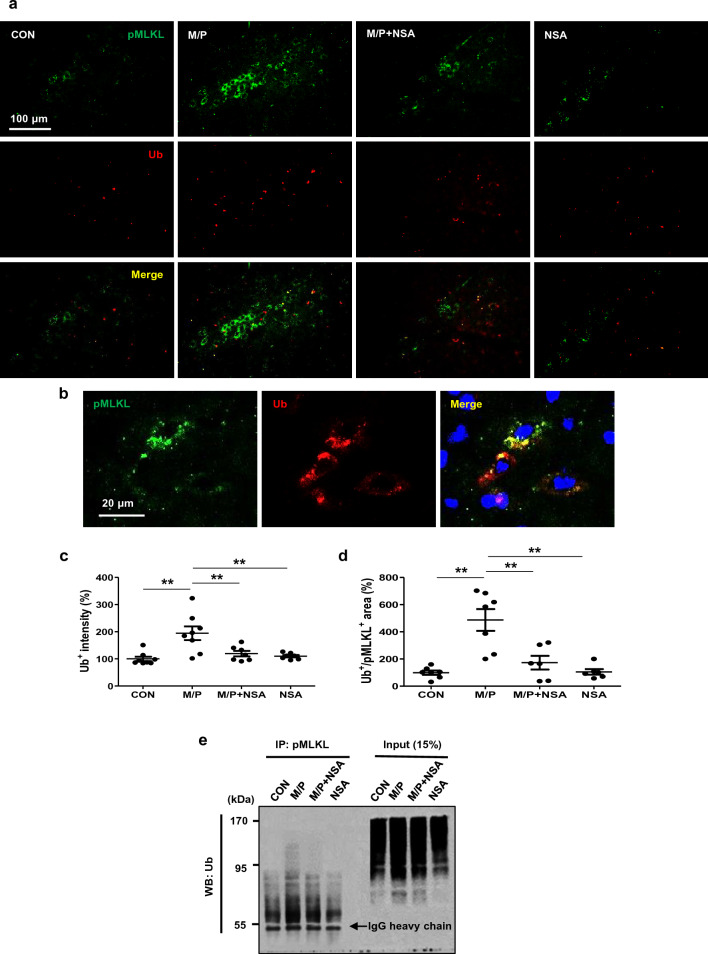
NSA diminished the ubiquitylation levels of pMLKL in MPTP-treated mice. (**a**) NSA diminished Ub^+^ punctae intensity and Ub^+^/pMLKL^+^ area in the SN of MPTP-treated mice. (**b**) A photomicrograph showing a high magnification of Ub^+^/pMLKL^+^ area. (**c**,**d**) A diagram quantifying the Ub^+^ punctae intensity and Ub^+^/pMLKL^+^ area (CON N = 8; M/P N = 8; M/P + NSA N = 7; NSA N = 7). (**e**) Ubiquitylated pMLKL was revalidated by co-immunoprecipitation assay (four samples per group were pooled: CON N = 4; M/P N = 4; M/P + NSA N = 4; NSA N = 4). Data are expressed as mean ± SEM. *p < 0.05, **p < 0.01.

Endogenous MLKL is ubiquitylated at some lysine residues, which is required for retaining the cell death-inducing activity of MLKL^13^. To examine the ubiquitylation state of phosphorylated MLKL protein and the impact of NSA in the MPTP-intoxicated SN area, double immunofluorescence staining with anti-pMLKL and anti-Ub antibodies was performed. Ub^+^ punctae intensity and Ub^+^/pMLKL^+^ area were significantly enhanced in the SN region of MPTP mice, and NSA significantly diminished both Ub^+^ intensity and Ub^+^/pMLKL^+^ area (Fig. 3a–d; for Ub^+^, F_3, 26_ = 8.67, *p* < 0.01; for Ub^+^/pMLKL^+^, F_3, 26_ = 13.86, *p* < 0.01). Furthermore, we verified that NSA suppressed ubiquitylated pMLKL levels by performing a co-immunoprecipitation assay (four samples per group were pooled; Fig. 3e). These results indicate that NSA reduces the ubiquitylation of p-MLKL, which is necessary for necroptotic execution in PD pathology.

### NSA reduced co-localization of active MLKL with p-α-synuclein in the SN region of MPTP-treated mice

**Figure 4:**
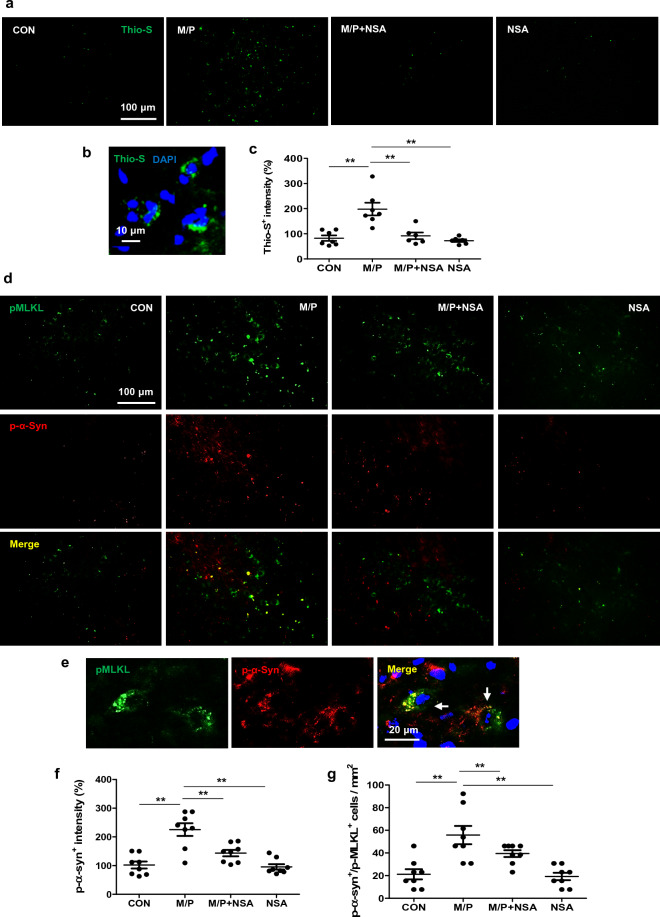
NSA inhibited the protein aggregates and decreased the number of cells co-expressing pMLKL and p-α-synuclein in MPTP-treated mice. (**a**) NSA reduced the thio-S^+^ punctae in the SN area of MPTP-treated mice. (**b**) The photomicrograph showing the high magnification of thio-S^+^ punctae. (**c**) A diagram quantifying the thio-S^+^ area (CON N = 7; M/P N = 7; M/P + NSA N = 6; NSA N = 6). (**d**) NSA attenuated p-α-synuclein^+^ punctae and p-α-synuclein^+^/pMLKL^+^ cells in the SN area of MPTP-treated mice. (**e**) A photomicrograph showing a high magnification of p-α-synuclein^+^/pMLKL^+^ cells. (**f**,**g**) A diagram showing the p-α-synuclein^+^ intensity and p-α-synuclein^+^/pMLKL^+^ cell quantification (CON N = 8; M/P N = 8; M/P + NSA N = 8; NSA N = 8). Data are expressed as mean ± SEM. ** *p* < 0.01.

Accumulated α-synuclein aggregates are a hallmark of PD, and the phosphorylated form of α-synuclein (Ser219-p) is particularly prone to aggregation. In this study, thioflavin-S (thio-S) staining revealed that MPTP treatment caused the deposition of protein aggregates, and NSA markedly attenuated thio-S^+^ punctae in the MPTP-intoxicated SN region (Fig. 4a–c; F_3, 22_ = 13.62, *p* < 0.01). To investigate the role of necroptosis in α-synuclein pathology, double immunofluorescence staining in the SN of MPTP mice was performed using antibodies against pMLKL and p-α-synuclein. A higher p-MKLK^+^/p-α-synuclein^+^ cell population was observed in MPTP-treated mice than in control mice, and NSA lowered the population (Fig. 4d–g; for p-MKLK^+^, F_3, 28_ = 16.59, *p* < 0.01; for p-MKLK^+^/p-α-synuclein^+^, F_3, 28_ = 11.20, *p* < 0.05). These findings suggest that α-synuclein aggregates may contribute to MLKL-dependent necroptotic signaling in PD progression, or vice versa.

### NSA reduced α-synuclein oligomerization by inhibiting the activities of GSK3β and metalloproteinase-3 (MMP3) in MPTP-treated mice

**Figure 5:**
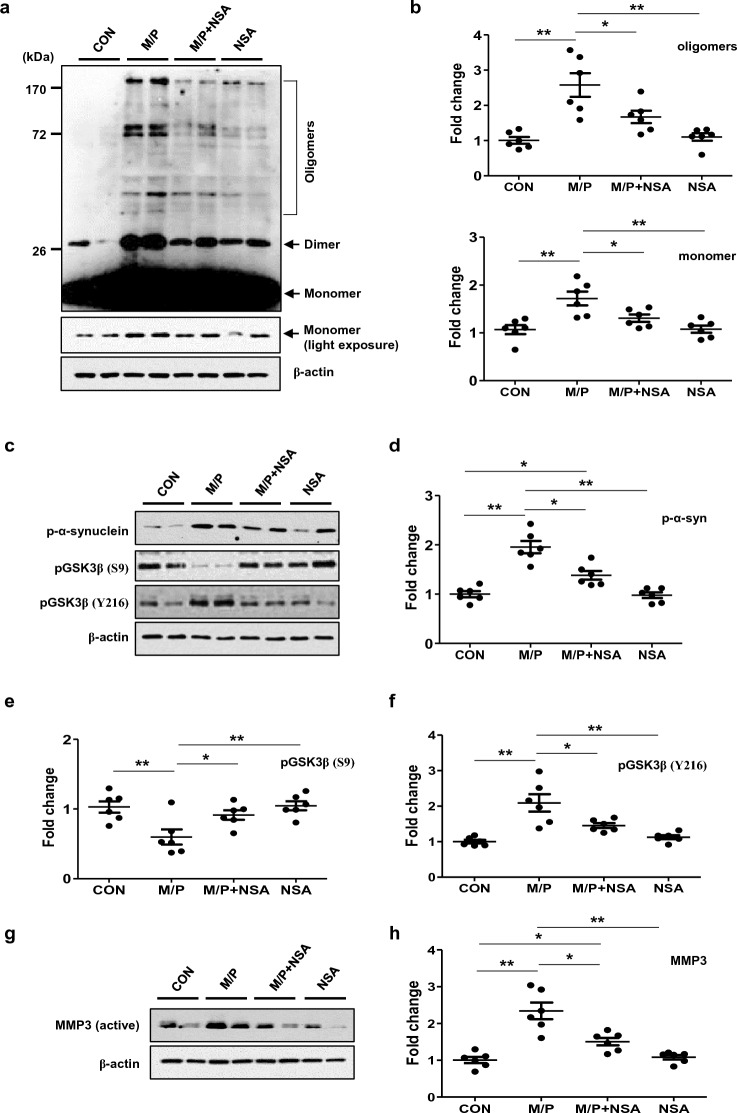
NSA suppressed α-synuclein oligomerization by inhibiting GSK3 activity and MMP3 expression in MPTP-treated mice. (**a**) NSA reduced α-synuclein monomer and oligomer levels in the SN area of the MPTP-treated mice. (**b**) A quantification diagram for α-synuclein monomer and oligomer levels (CON N = 6; M/P N = 6; M/P + NSA N = 6; NSA N = 6). (**c**) NSA downregulated p-α-synuclein and pGSK3β (Y216-p) levels, and upregulated pGSK3β (S9-p) levels in the SN area of the MPTP-treated mice. (**d**–**f**) A quantification diagram for p-α-synuclein, pGSK3β (Y216-p), and pGSK3β (S9-p) levels (CON N = 6; M/P N = 6; M/P + NSA N = 6; NSA N = 6). (**g**) NSA reduced MMP3 levels in the SN area of MPTP-treated mice. (**h**) A quantification diagram of the MMP3 levels (CON N = 6; M/P N = 6; M/P + NSA N = 6; NSA N = 6). Data was expressed as mean ± SEM. *p < 0.05, **p < 0.01.

To investigate the potential role of MLKL in α-synuclein pathology further, we examined the effect of NSA on α-synuclein expression and oligomerization. Western blot analysis revealed that NSA decreased the expression of α-synuclein monomers and oligomers in the SN region of MPTP mice (Fig. 5a,b; monomer, F_3, 20_ = 9.13, *p* < 0.01; oligomers, F_3, 20_ = 12.66, *p* < 0.01). Additionally, NSA treatment reduced p-α-synuclein levels (Fig. 5c,d; F_3, 20_ = 27.60, *p* < 0.01). Next, we examined the effects of NSA on GSK3β and MMP-3, which are involved in α-synuclein aggregation. The activity of GSK3β depends on its phosphorylation site (at Tyr216; kinase-active, at Ser9; kinase-inhibitory), which plays a crucial role in α-synuclein phosphorylation^35,36^. NSA treatment shifted from MPTP-induced kinase-active (Tyr216-p) GSK3β to kinase-inhibitory (Ser9) in the SN area (Fig. 5c,e,f; Tyr216-p, F_3, 20_ = 13.69, *p* < 0.01; Ser9-p, F_3, 20_ = 6.34, *p* < 0.01). Furthermore, NSA inhibits the expression of MMP-3, which cleaves α-synuclein into aggregate-prone species^37,38^. (Fig. 5g,h; F_3, 20_ = 20.70, *p* < 0.01). These findings suggest that NSA inhibits α-synuclein oligomerization by modulating the expression of GSK3β and MMP3.

### NSA suppressed neuroinflammation in MPTP-treated mice

**Figure 6:**
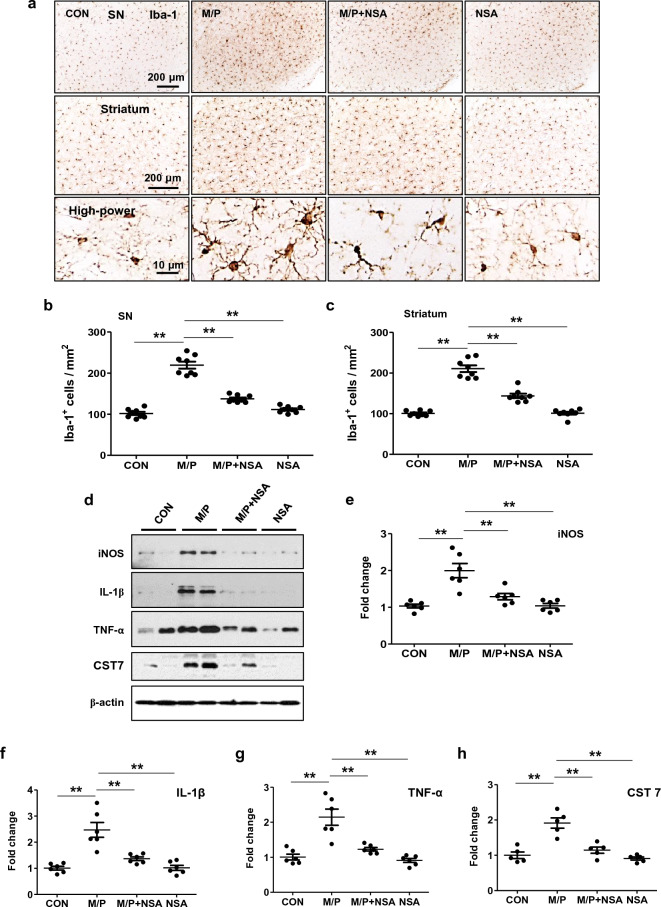
NSA inhibited microglial activation and the expression of proinflammatory mediators in MPTP-treated mice. (**a**) NSA reduced the number of Iba-1^+^ microglial cells in the striatum and the SN of MPTP-treated mice. (**b**,**c**) A diagram quantifying the Iba-1^+^ cells in the SN (**b**; CON N = 8; M/P N = 8; M/P + NSA N = 8; NSA N = 8) and the striatum (**c**; CON N = 8; M/P N = 8; M/P + NSA N = 8; NSA N = 8). (**d**) The NSA downregulated the levels of iNOS, IL-1β, TNF-α, and CST7 in the SN of MPTP-treated mice. (**e**–**h**) A quantification diagram for iNOS, IL-1β, TNF-α, and CST7 levels (CON N = 6; M/P N = 6; M/P + NSA N = 6; NSA N = 6). Data was presented as mean ± SEM. *p < 0.05, **p < 0.01.

As neuroinflammation is a crucial event in PD pathogenesis and necroptosis, we examined the effect of NSA on inflammatory responses in MPTP mice. According to immunohistological analysis, NSA inhibited microglial activation in the striatum and SN of MPTP mice (Fig. 6a–c; SN, F_3, 28_ = 10.95, *p* < 0.01; striatum, F_3, 28_ = 11.35, *p* < 0.01). Furthermore, NSA inhibited the expression of proinflammatory markers induced by MPTP, such as iNOS, IL-1β, and TNF-α (Fig. 6d–g; iNOS, F_3, 20_ = 16.22, *p* < 0.01; IL-1β, F_3, 20_ = 19.25, *p* < 0.01; TNF-α, F_3, 20_ = 19.35, *p* < 0.01). Moreover, NSA inhibited the expression of cystatin F (CST7), a key modulator of inflammatory responses in microglia^39,40^ (Fig. 6d,h; F_3, 16_ = 21.06, *p* < 0.01). These results indicate that NSA possesses potent anti-inflammatory properties.

### NSA suppressed reactive astrogliosis in MPTP-treated mice

**Figure 7:**
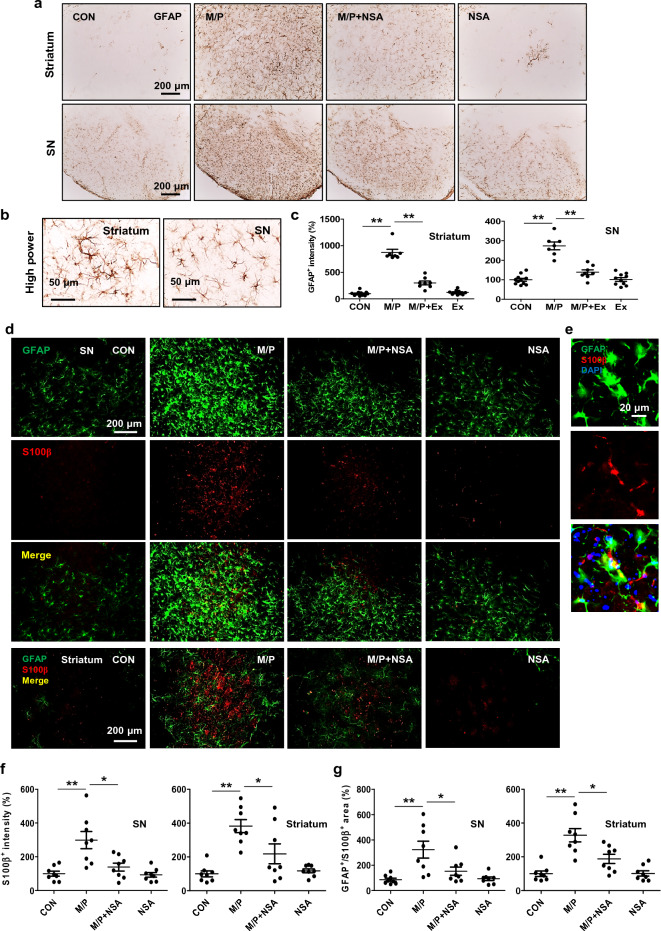
NSA suppressed reactive astrogliosis and S100β expression in MPTP-treated mice. (**a**) NSA dampened the GFAP^+^ intensity in the striatum and SN of MPTP-treated mice. (**b**) Photomicrograph showing the high magnification of GFAP^+^ punctae. (**c**) A diagram quantifying the GFAP^+^ intensities in the SN and striatum (CON N = 11; M/P N = 7; M/P + NSA N = 8; NSA N = 10). (**d**) NSA attenuated the S100β^+^ intensity and S100β^+^/GFAP^+^ area in the striatum and SN of MPTP-treated mice. (**e**) Photomicrograph showing the high magnification of S100β^+^/GFAP^+^ area. (**f**,**g**) Diagram showing the S100β^+^ intensity and S100β^+^/GFAP^+^ area quantification (CON N = 8; M/P N = 8; M/P + NSA N = 8; NSA N = 8). Data are presented as mean ± SEM. *p < 0.05, **p < 0.01.

**Figure 8:**
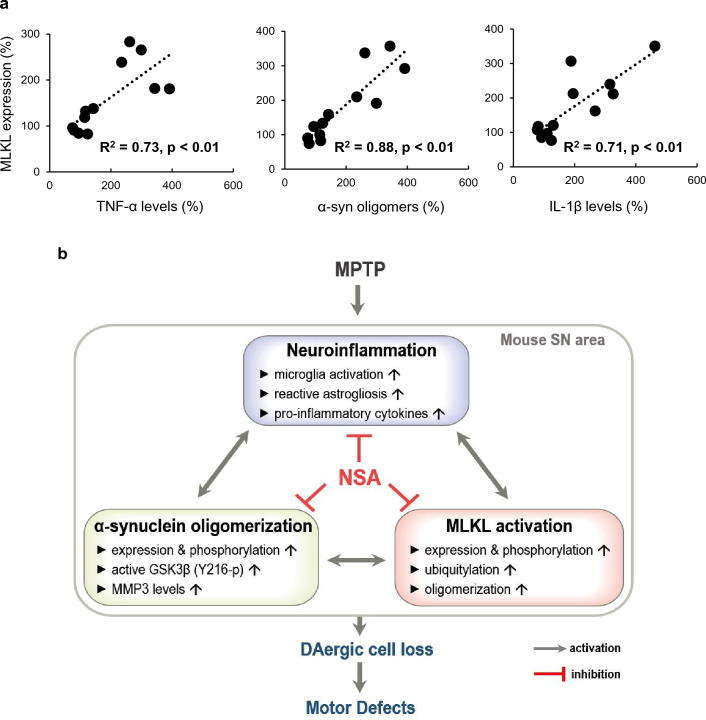
Proposed mechanism underlying the neuroprotective effect of NSA in an MPTP-induced PD mouse model. (**a**) Under MPTP-cytotoxic conditions, TNF-α, α-synuclein oligomers, and IL-1β levels are positively correlated with MLKL expression in vivo. (**b**) A possible mechanism for the neuroprotective role of NSA. MPTP causes pathological changes in the mouse SN area, including neuroinflammation, α-synuclein oligomerization, and MLKL activation. The reciprocal interaction between each detrimental factor may contribute to exacerbating dopaminergic degeneration. However, NSA has potent anti-inflammatory and anti-α-synucleinopathic effects in the SN area, as well as anti-necroptotic effect. These beneficial effects of NSA may result in neurorestoration against MPTP-neurotoxicity in a mouse model of PD.

We examined the effect of NSA on astrocyte reactivity using GFAP and S100β staining in MPTP mice as reactive astrogliosis occurs during PD progression. NSA attenuated the MPTP-induced enhancement of GFAP^+^ intensity in the SN and striatum (Fig. 7a–c; SN, F_3, 32_ = 43.16, *p* < 0.01; striatum, F_3, 32_ = 126.83, *p* < 0.01) indicating that NSA suppressed reactive astrogliosis in MPTP-treated mice. The S100β protein is predominantly derived from astrocytes in the CNS. Excessive production of S100β is known to activate RAGE-dependent inflammation and produce ROS, causing parenchymal cell damages in the brain^41,42^. In this study, we found that NSA attenuated the MPTP-induced augmentation of S100β^+^ intensities and S100β^+^/GFAP^+^ area in the SN and striatum (Fig. 7d–g; SN—for S100β^+^ intensity, F_3, 28_ = 10.55, *p* < 0.01; for S100β^+^/GFAP^+^ area, F_3, 28_ = 8.38, *p* < 0.01; striatum—for S100β^+^ intensity, F_3, 28_ = 12.47, *p* < 0.01; for S100β^+^/GFAP^+^ area, F_3, 28_ = 16.45, *p* < 0.01). These results indicate that NSA suppresses MPTP-induced astrogliosis in the nigrostriatal dopaminergic circuit. Based on our experimental paradigm, we examined the statistical relationship in which each variable moves in coordination with one another using Pearson’s correlation. Under MPTP-cytotoxic conditions, TNF-α (necroptotic trigger), α-synuclein oligomers (α-synuclein pathology), and IL-1β (inflammation marker) levels were strongly correlated with MLKL expression (necroptosis executor) (Fig. 8a; for TNF-α, R^2^ = 0.73, *p* < 0.01; for α-synuclein oligomers, R^2^ = 0.88, *p* < 0.01; for IL-1β, R^2^ = 0.71, *p* < 0.01).

## Discussion

PD causes motor performance defects due to basal ganglion dysfunction, which is characterized by dopaminergic cell death, neuroinflammation, and α-synucleinopathy. Heterogeneous cell death, such as apoptosis and necroptosis, occurs during PD progression^22,23,43,44^. Necroptosis is an inflammatory form of programmed cell death tightly regulated by the RIPK1/RIPK3/MLKL axis. As MLKL is an executor in necroptotic signaling, targeting MLKL has been suggested as an attractive strategy for restraining the pathological progression of PD. In this study, we tested the therapeutic efficacy of NSA, a pharmacological inhibitor of MLKL, in a mouse model of PD. NSA restored motor coordination and akinesia, and reversed dopaminergic cell loss in the SN area and the blunted neurotransmission toward the striatum. These results suggest the involvement of necroptosis in dopaminergic cell loss and the protective role of NSA in mice with PD.

PTMs of MLKL, including phosphorylation and ubiquitylation, are required for its cell lysis-inducing activity^11–13^. RIPK3-dependent phosphorylation at Ser345, Ser347, and Thr349 in the pseudokinase domain within the C-terminal region of murine MLKL occurs upon necroptotic stimulation, and MLKL phosphorylation at Ser345 promotes oligomerization, facilitating membrane targeting^45–47^. MLKL is conjugated to the K63-linked ubiquitin chain under necroptotic signaling, and ubiquitylation of MLKL at Lys219 induces optimal oligomerization at the plasma membrane and augments MLKL activity to potentiate cytotoxic effects^11,13^. This study revealed more pMLKL^+^/TH^+^ and pMLKL^+^/Ub^+^ cells measured through histological data, and higher ubiquitylated pMLKL levels measured using co-immunoprecipitation assay in MPTP-intoxicated SN than in the control group, and the reversal effects of NSA exposure implied that repeated treatment with NSA hinders the cell death-inducing activity of MLKL in PD mice. Furthermore, our finding that NSA had no effect on the MPTP-induced increase in RIPK1 and RIPK3 protein levels corresponded to previous findings in rodent models^4,31^, indicating that NSA acts specifically on MLKL in vivo. NSA treatment reduced the MPTP-induced increase in MLKL protein levels in the MPTP mice. In protein homeostasis, functional proteins are retained in their proper contents and cellular compartments, where misfolded, damaged, or redundant proteins undergo protein degradation via proteolytic machinery^48^. Moreover, active MLKL reportedly forms amyloid-like polymers and attenuates autophagic flux, rendering MLKL resistant to protease digestion^49,50^. In this study, we demonstrated that NSA reduced MLKL tetramer levels in the insoluble fraction and MLKL oligomer formation in the SN of MPTP-treated mice. Based on previous findings, the inhibition of active MLKL by NSA may promote protein turnover and block the further expansion of necroptotic signaling.

The study’s main finding was that NSA inhibits α-synuclein oligomerization and neuroinflammation in a mouse model of PD. Some research has suggested a link between α-synuclein aggregation and neuroinflammation in PD pathology, in which α-synuclein oligomers activate microglia via toll-like receptor 2, and reactive oxygen species released by activated microglia facilitate α-synuclein aggregation^51–53^. On the other hand, necroptosis can trigger inflammation via the release of proinflammatory factors such as DAMPs^5–7^. Moreover, active MLKL induces NLRP3 inflammasome activation, leading to secretion of IL-1β^1^. In this study, NSA-mediated inhibition of active MLKL alleviated neuroinflammation in the MPTP-intoxicated SN area, as indicated by decreased microglial reactivity and proinflammatory mediators such as iNOS, IL-1β, TNF-α, and CST7. Moreover, the detrimental role of reactive astrocytes in PD pathogenesis is reportedly associated with overexpression and release of S100β in PD animal models and patients with PD^54–56^. In this study, NSA suppressed reactive astrogliosis in MPTP-intoxicated SN and striatum, as indicated through reduced S100β^+^ intensity and S100β^+^/GFAP^+^ area. These results imply that NSA ameliorates microglial activation- and reactive astrogliosis-mediated neuroinflammation, thereby restoring neurodegenerative phenotypes. Furthermore, NSA inhibited the MPTP-induced expression of active GSK3β and MMP3, which are increased under neuroinflammatory conditions^33,57,58^. Thus, by inhibiting GSK3β and MMP3 activity, NSA’s anti-inflammatory effects may contribute to the relief of α-synuclein pathology. Previous research and our findings suggest that repeated NSA administration may disrupt the forward feed loop in neuroinflammation, α-synuclein pathology, and MLKL activation in the MPTP mouse model of PD. The proposed mechanism of the NSA is shown in Fig. 8b.

In conclusion, this is the first study to show that NSA has a therapeutic effect on PD-like behavioral defects and pathophysiology, such as necroptosis, neuroinflammation, and α-synuclein oligomerization, in a subacute MPTP mouse model of PD. Consequently, our findings add to the evidence that necroptosis plays a role in PD pathogenesis and support the anti-parkinsonian effect of NSA.

## Supplementary Information

Supplementary Information 1.

Supplementary Information 2.

## Data Availability

The datasets used and/or analyzed during the current study are available from the corresponding author on reasonable request.

## Abbreviations

AD: Alzheimer’s disease
CST7: Cystatin F
DAMP: Damage-associated molecular pattern
GSK3β: Glycogen synthase kinase 3beta
IL-1β: Interleukin-1beta
iNOS: Inducible nitric oxide synthase
MMP3: Matrix metalloproteinase-3
MLKL: Mixed lineage kinase domain-like protein
MPTP: 1-Methyl-4-phenyl-1,2,3,6-tetrahydropyridine
MPTP/P: MPTP/probenecid
Nec-1: Necrostatin-1
Nec-1s: Nec-1 stable
NSA: Necrosulfonamide
6-OHDA: 6-Hydroxydopamine
RIPK: Receptor-interacting protein kinase
PD: Parkinson’s disease
PTM: Post-translational modification
SN: Substantia nigra
Thio-S: Thioflavin-S
TNF-α: Tumor necrosis factor-alpha
Ub: Ubiquitin
