# Supplementary material for: Necrosulfonamide exerts neuroprotective effect by inhibiting necroptosis, neuroinflammation, and α-synuclein oligomerization in a subacute MPTP mouse model of Parkinson’s disease
Source: Sci Rep. 2023 May 31;13:8783. doi: 10.1038/s41598-023-35975-y (PMC10232437; doi:10.1038/s41598-023-35975-y)
Supplement: Supplementary file 1 — Supplementary Information 1. [file 41598_2023_35975_MOESM1_ESM.pdf]

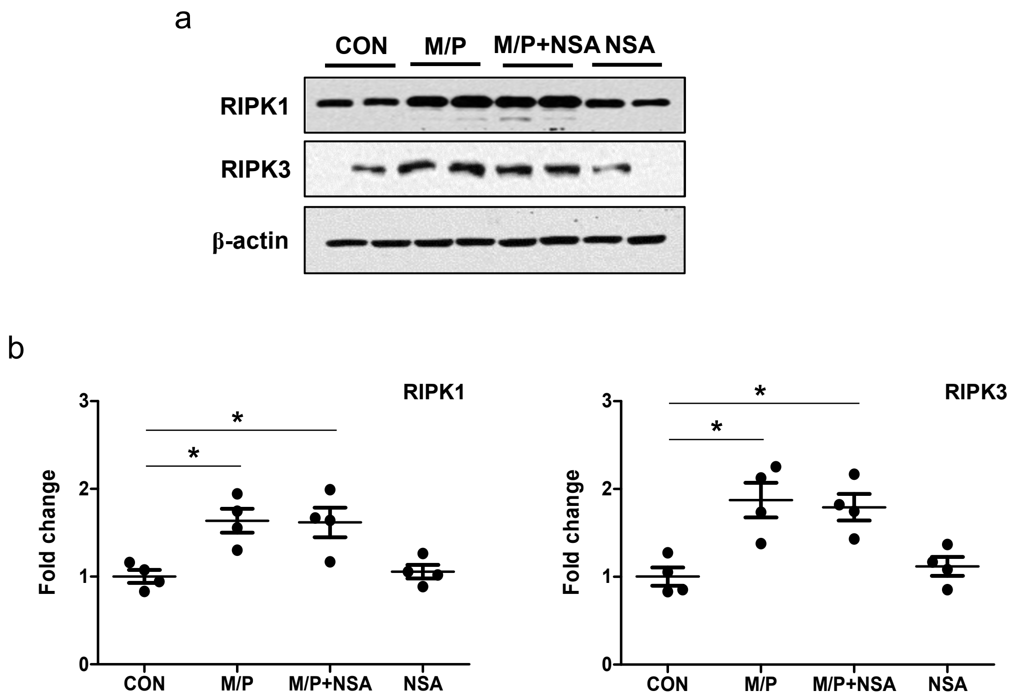

Supplementary Figure 1. NSA did not affect the expression of RIPK1 and RIPK3 proteins in MPTP-treated mice. (a) Western blot analysis of the SN region shows that MPTP treatment increased the expression levels of RIPK1 and RIPK3 proteins compared to control mice. However, the RIPK1/3 levels induced by MPTP were not altered by NSA treatment. (b) A quantification diagram of the RIPK1 and RIPK3 protein levels (for RIPK1, $F_{3, 12} = 8.16$; for RIPK3, $F_{3, 12} = 9.61$; N = 4 for each group). * denotes $p < 0.05$.
