# Supplementary material for: Necrosulfonamide exerts neuroprotective effect by inhibiting necroptosis, neuroinflammation, and α-synuclein oligomerization in a subacute MPTP mouse model of Parkinson’s disease
Source: Sci Rep. 2023 May 31;13:8783. doi: 10.1038/s41598-023-35975-y (PMC10232437; doi:10.1038/s41598-023-35975-y)

# Original Images of Uncropped Blots

## [Fig. 2a; uncropped blots]

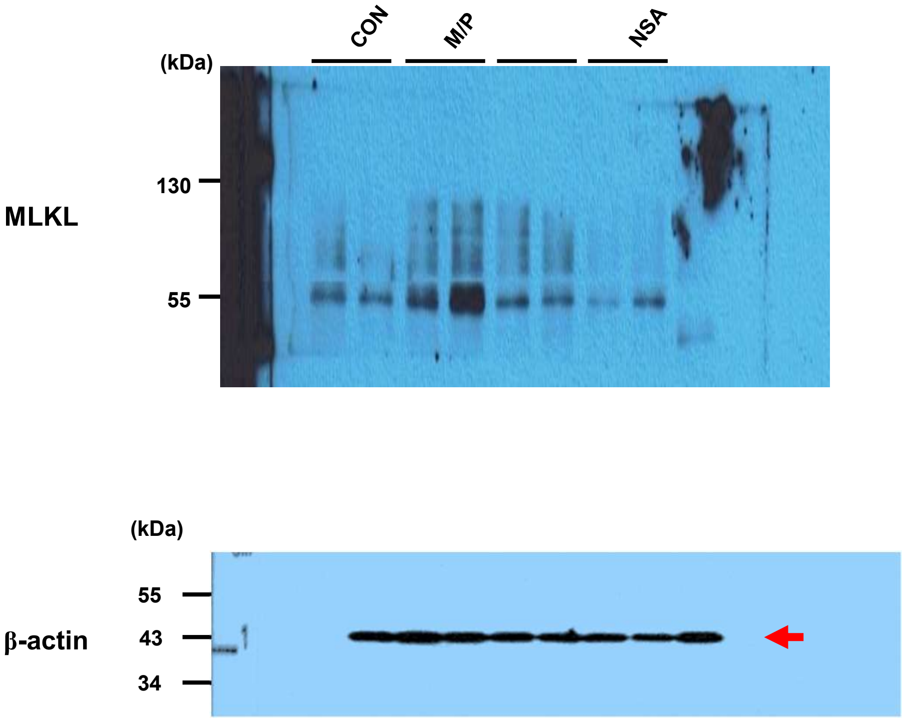


## [Fig. 2c; uncropped blot]

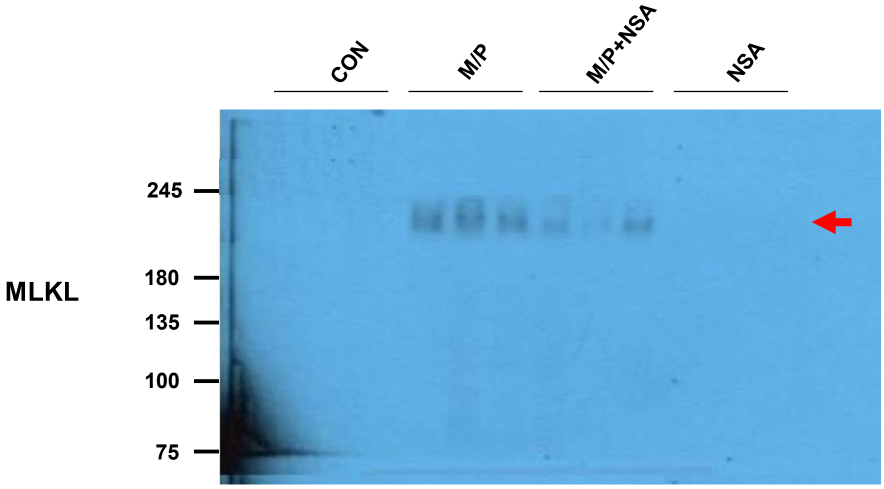

## [Fig. 2e; uncropped blots]

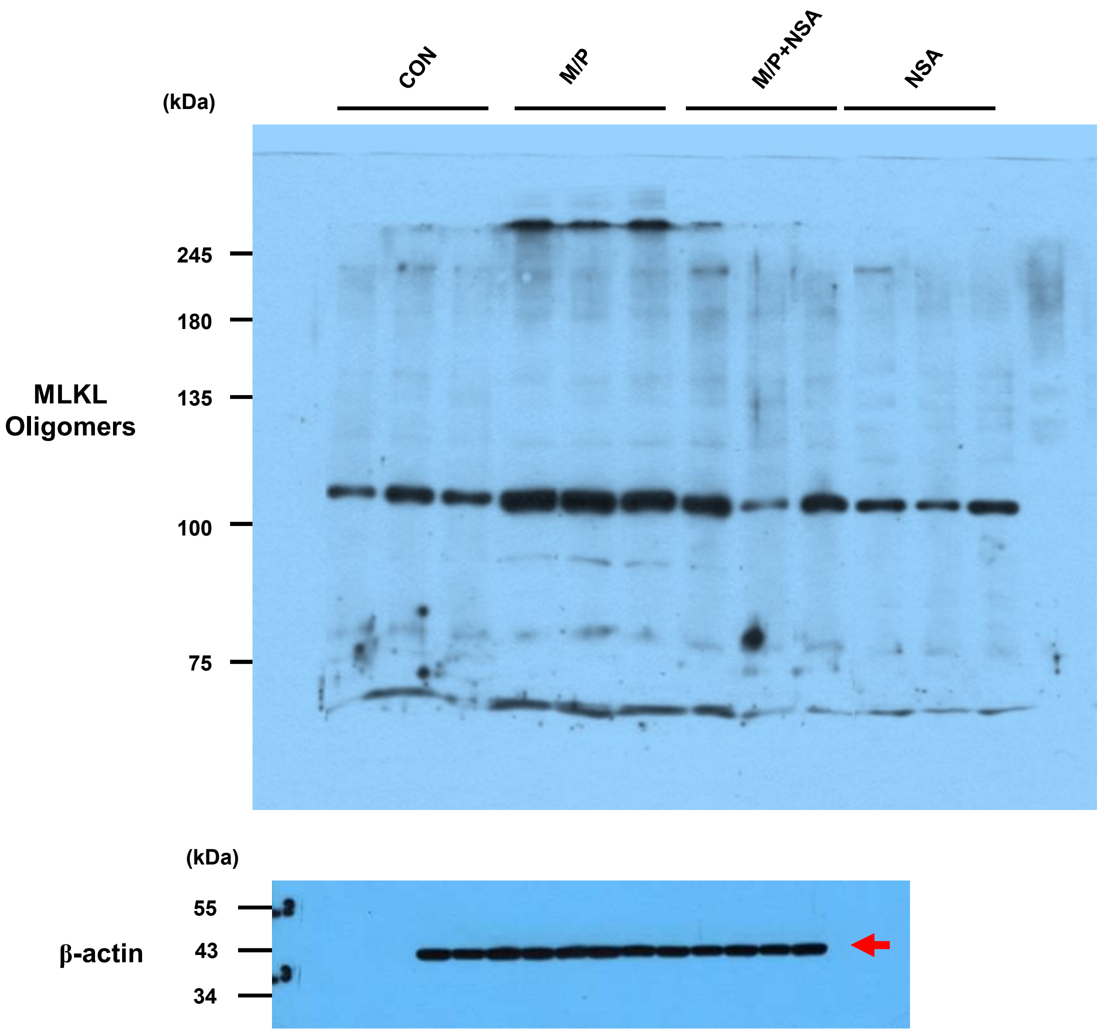

# [Fig. 3e; uncropped blot]

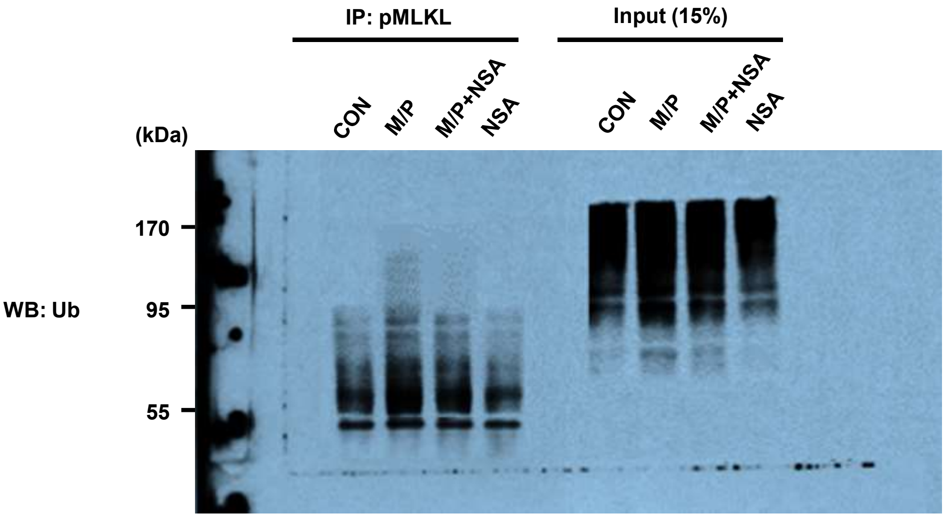

## [Fig. 5a; uncropped blot]

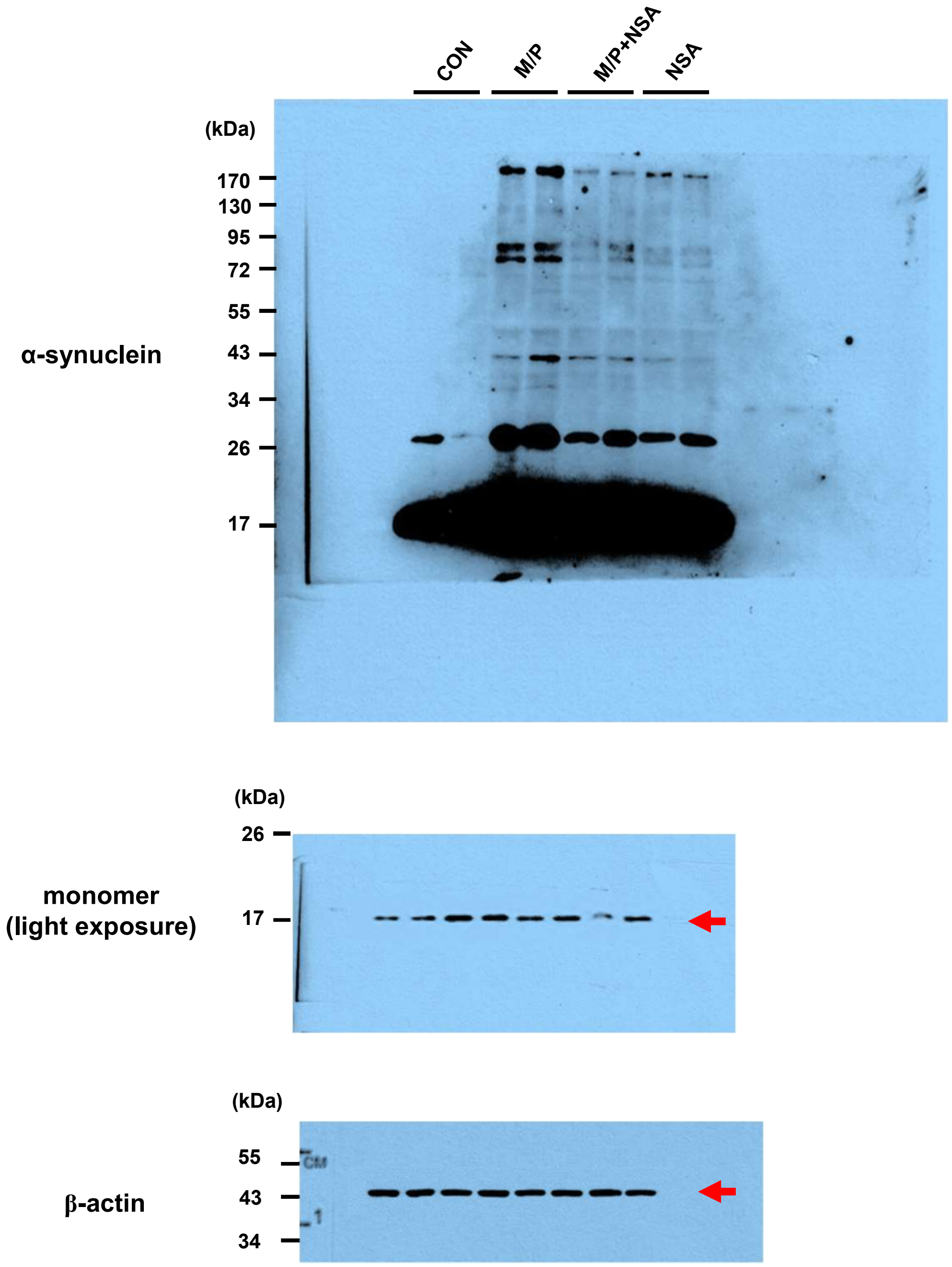

# [Fig. 5c, g; uncropped blots]

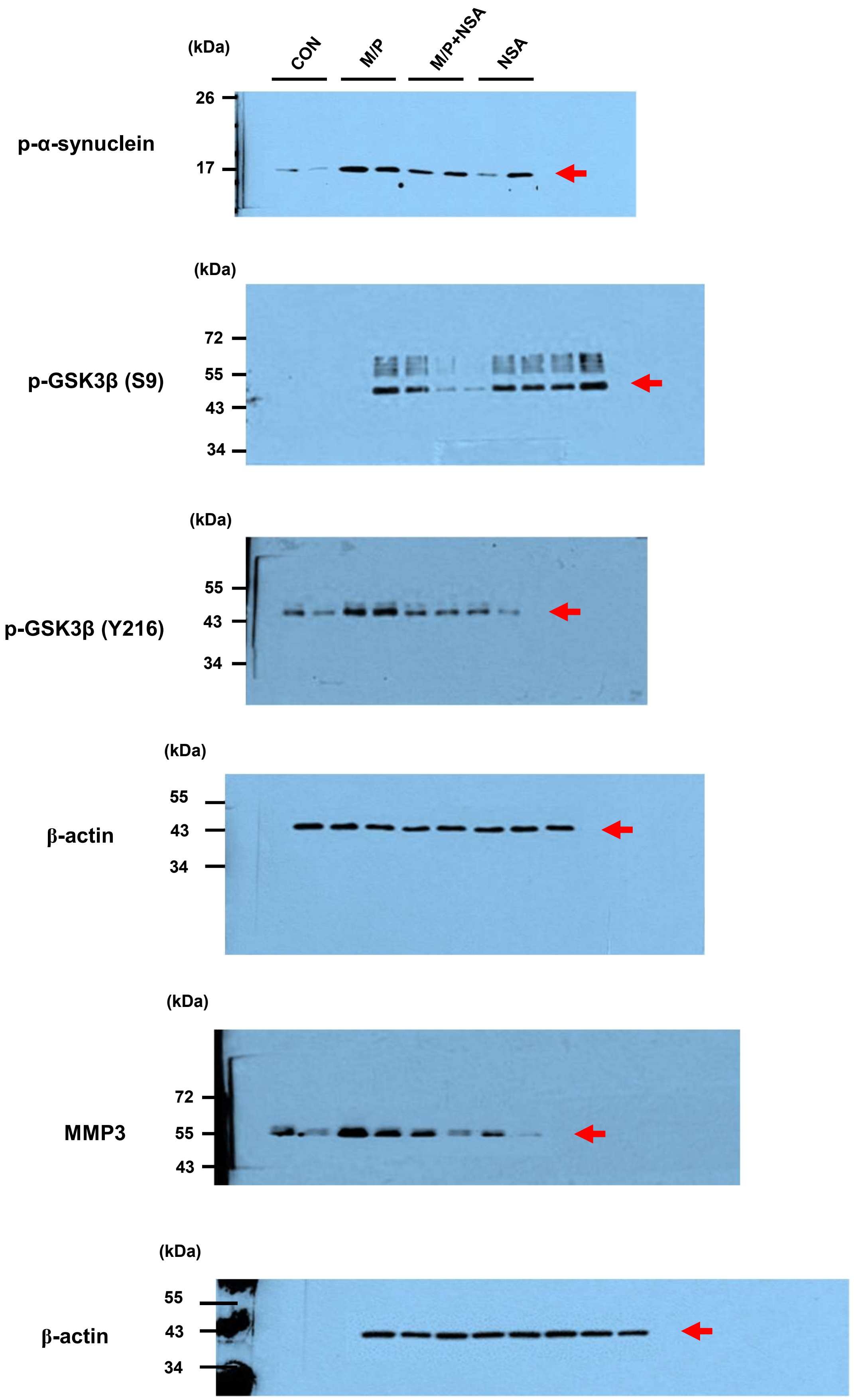

**[Fig. 6d; uncropped blots]**

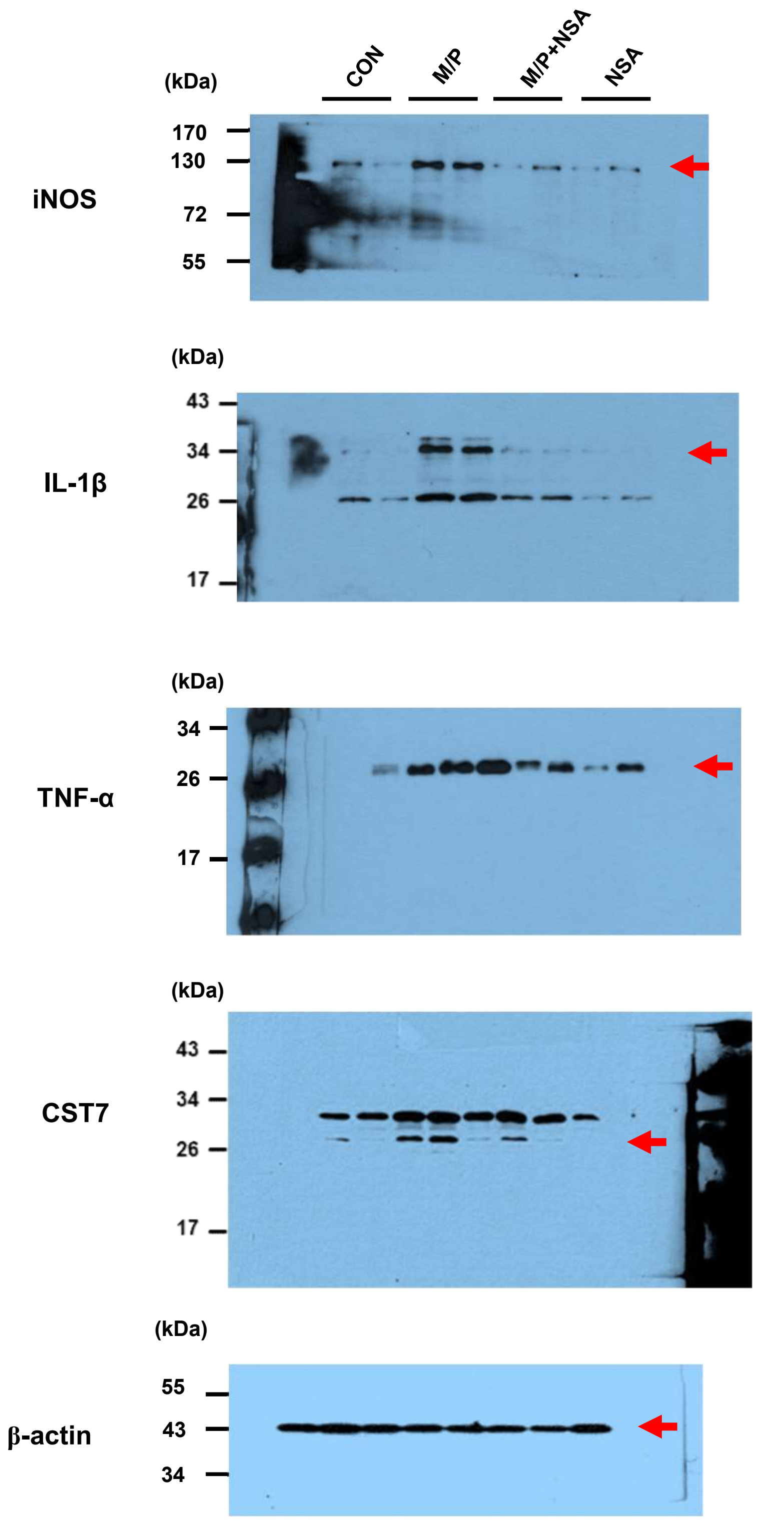

# [Suppl Fig. 1a; uncropped blots]

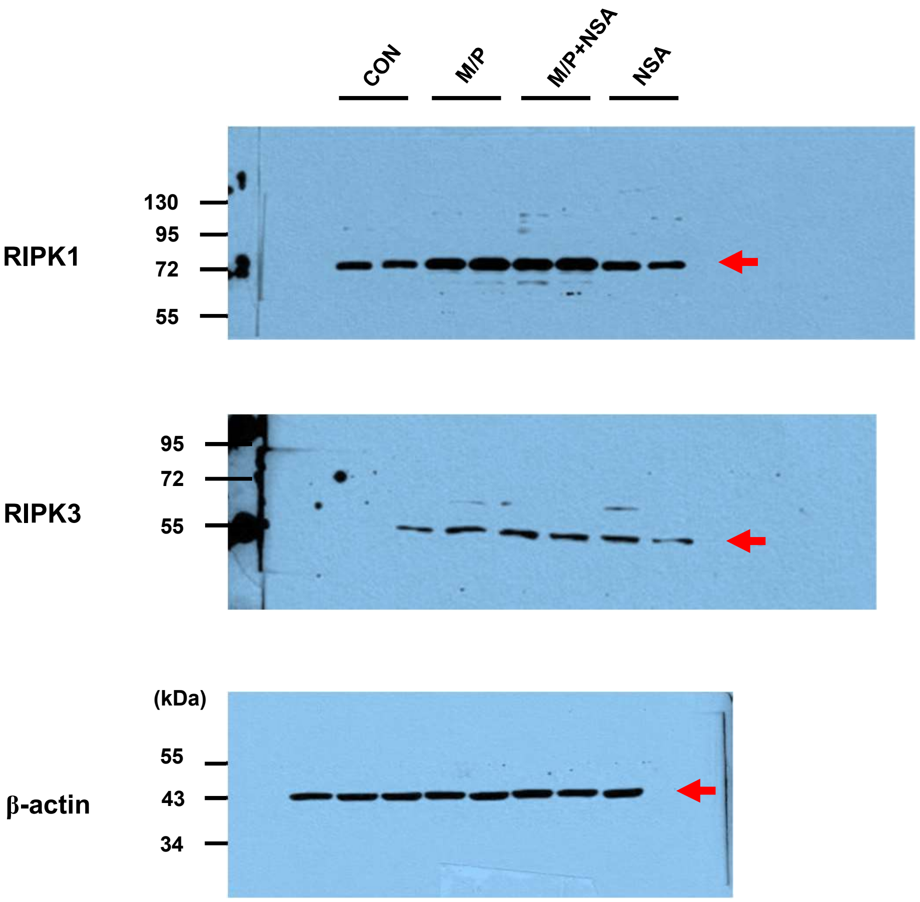

Supplement: Supplementary file 2 — Supplementary Information 2. [file 41598_2023_35975_MOESM2_ESM.pdf]
